# Development of an infant colon simulating *in vitro* model, I-TIM-2, to study the effects of modulation strategies on the infant gut microbiome composition and function

**DOI:** 10.1128/spectrum.00724-24

**Published:** 2024-10-08

**Authors:** Olivia Colberg, Gerben D. A. Hermes, Tine Rask Licht, Anita Wichmann, Adam Baker, Martin Frederik Laursen, Anja Wellejus

**Affiliations:** 1 Novonesis, Human Health Research, Hørsholm, Denmark; 2 Technical University of Denmark, National Food Institute, Lyngby, Denmark; 3 Chr. Hansen, Human Health Research, Hørsholm, Denmark; University of Nebraska-Lincoln, Lincoln, Nebraska, USA

**Keywords:** infant, colonic model, microbiome, metabolome

## Abstract

The early life stages are critical for the development of the gut microbiome. Variables such as antibiotics exposure, birth-mode via Cesarean section, and formula feeding are associated with disruptions in microbiome development and are related to adverse health effects later in life. Studying the effects of microbiome-modulating strategies in infants is challenged by appropriate ethical constraints. Therefore, we developed I-TIM-2, an infant *in vitro* colonic model based on the validated, computer-controlled, dynamic model of the colon, TIM-2. The system, consisting of four separate compartments, was inoculated with feces from four healthy, primarily breastfed infants, displaying distinctive microbiome profiles. For each infant’s fecal sample, a 96-h experiment was performed, with two compartments receiving an infant diet adapted medium and two compartments additionally receiving five human milk oligosaccharides (HMOs) in physiological concentrations and proportions. Bacterial composition was determined by shotgun metagenomics and qPCR. Concentrations of short-chain fatty acids (SCFAs) and HMOs were determined by LC-MS. Microbial diversity and high amounts of inoculum-derived species were preserved in the model throughout each experiment. Microbiome composition and SCFA concentrations were consistent with published data from infants. HMOs strongly modulated the microbiome composition by stimulating relative proportions of *Bifidobacterium*. This affected the metabolic output and resulted in an increased production of acetic and formic acid, characteristic of bifidobacterial HMO metabolism. In conclusion, these data demonstrate the development of a valid model to study the dynamics and modulations of the infant gut microbiome and metabolome.

**IMPORTANCE:**

The infant gut microbiome is intricately linked to the health of its host. This is partly mediated through the bacterial production of metabolites that interact with the host cells. Human milk shapes the establishment of the infant gut microbiome as it contains human milk sugars that select for primarily bifidobacteria. The establishment can be disrupted by modern interventions such as formula feeding. This can alter the microbiome composition and metabolite production profile, which can affect the host. In this article, we set up an infant *in vitro* colonic model to study microbiome interactions and functions. In this model, we investigated the effects of human milk sugars and their promotion of bifidobacteria at the expense of other bacteria. The model is an ideal system to assess the effects of various modulating strategies on the infant gut microbiome and its interactions with its host.

## INTRODUCTION

The human gut microbiome contains up to trillions of microbes of which bacteria make up the vast majority (1). It impacts host physiology and development, and is essential to lifelong health (2). Colonization begins at birth with the infant receiving the earliest microbes through vertical transmission from its mother and the environment (3).

Early infancy represents an essential time period for the establishment of the gut microbiome. Perturbations caused by factors such as formula feeding, birth mode via Cesarean section, preterm birth, and antibiotics use have been associated with adverse health outcomes in later life. These include obesity (4), chronic gastrointestinal disorders (5), metabolic diseases (6), and autoimmune diseases such as asthma (7) and allergies (8). Therefore, there has been increasing interest in exploring microbiome-modulating strategies in infancy to ensure favorable microbial succession patterns and host interactions (9).

However, investigating microbiome-modulating strategies in infants is challenging. Clinical studies are appropriately constrained by ethical and regulatory restrictions to protect this particularly sensitive population group (10, 11). In addition, conclusions from animal models cannot be directly extrapolated to humans, and legislation strives to decrease the use of animal experimentation (12). Therefore, *in vitro* colonic models that simulate the infant colonic environment are valuable tools for studying the infant gut microbiome. They allow for frequent sampling, modeling of disease states, and opportunities to screen many substances and biotherapeutics. In recent years many types of colonic *in vitro* models have been developed, ranging from static batch fermentation systems to more complex and dynamic models (13). Some have been set up to simulate infant conditions (14–20). Infants are distinct from adults in terms of diet and digestion (21). Thus, *in vitro* models set up for this particular environment are needed to obtain meaningful observations and conclusions that apply to infants.

The infant gut microbiome is characterized by low diversity and large interindividual variation (22). Species of *Bifidobacterium* and *Bacteroides* are among the colonizers, as they can efficiently utilize human milk oligosaccharides (HMOs) (23, 24). HMOs are highly abundant in human milk (25) and are not digested by the infant, thus the majority reach the colon where they selectively promote the growth of HMO utilizing bacteria (26). Certain *Bifidobacterium* species, such as *B. bifidum*, *B. breve* and *B. longum s*ubsp. *longum* (*B. longum*) and *B. longum* subsp. *infantis* (*B. infantis*) and *Bacteroides* species such as *B. fragilis* and *B. thetaiotaomicron* degrade HMOs in a species and strain-dependent manner (27, 28).

The infant gut microbiome increases in diversity over time until childhood (29), where it becomes relatively stable and resilient to disturbances (30). Due to the lower bacterial density, diversity, and stability, early infancy is considered a “window of opportunity” for gut microbial modulation.

In this study, we set up an infant *in vitro* colonic model in TIM-2 (TNO *In vitro* Model). This is a validated, dynamic, and computer-controlled colon simulating model (31, 32), which has previously been used in experiments where the model was inoculated with fecal samples of human or animal origin. The model has mostly been used for simulation of human adult colonic conditions (33, 34), though recently it was also set up to simulate infant colonic conditions (20).

Here, we optimized the TIM-2 to simulate the infant colonic environment. The model, hereafter named Infant TIM-2 (I-TIM-2) was inoculated with fecal samples from four primarily breastfed infants to study the microbiome and metabolome with and without the addition of HMOs. We assessed the model’s ability to reproduce the composition, function, and metabolic output of the fecal bacterial communities and studied the effects of diet (formula feeding vs breastfeeding). For this, we used two media: a simulated infant ileal efflux medium (SIIEM), and SIIEM with an HMO mix (SIIEM-HMO) consisting of five abundant HMOs in human milk [2′-Fucosyllactose (2′-FL), 3-Fucosyllactose (3-FL), Lacto-N-Tetraose (LNT), 3′-Sialyllactose (3′-SL), and 6′-Sialyllactose (6′-SL)] in physiologically relevant ratios and concentrations.

This five-HMO combination, which represents some of the most abundant HMOs from each of the three structural classes (fucosylated, neutral core, and sialylated), was recently evaluated in a clinical study. Here, it was shown to promote a microbiome composition in formula-fed infants that was more similar to that of a breastfed reference group (35, 36).

## MATERIALS AND METHODS

### Infant fecal sample collection

Fecal samples from four healthy, Danish, 2- to 4-month-old infants (INF1–4) were used as inoculation material in four independent experiments. The infants were either exclusively breastfed or primarily breastfed and supplemented with infant formula feeding. Written permission was obtained from all parents. Fecal samples were collected from diapers and subsequently transferred and stored individually in sterile vials containing a dialysate solution (described in the next paragraph) with glycerol (10% vol/vol final concentration) as a cryoprotective agent, as described in reference (37). The samples were collected over a 6-week period to obtain adequate amounts of material. The fecal-dialysate mixtures were stored at the homes of the participants at −20°C, until they were transported on dry ice to the lab and stored at −80°C until use. Information on home environment, diet, and health status of the mothers and infants was recorded biweekly via questionnaires (Table 1). Data and samples were handled and stored according to applicable GDPR practices.

**TABLE 1:** Infant donor characteristics

Donor ID	Sex	Age (months)	Birth mode	Feeding mode
INF1	Female	3	Cesarian section	Exclusively breastfed
INF2	Male	2	Cesarian section	Mostly breastfed/some formula
INF3	Female	3	Vaginal	Mostly breastfed/some formula
INF4	Male	4	Vaginal	Exclusively breastfed

### Culture medium and dialysate solution

The standard TIM-2 culture medium was adapted to Simulated Infant Ileal Efflux Medium, SIIEM, to reflect the diet and physiology of infants and represent infant ileal efflux as previously described (38, 39). Bile salts, yeast extract, peptone, and tryptone were reduced to reflect the lower levels measured in infants compared to adults and complex carbohydrates were replaced with lactose, casein, and whey in concentrations based on the World Health Organization’s official guidelines on infant formula feeding (150 mL milk kg^−1^ bodyweight per 24 h, using the average body weight of 4- to 6-month-old infants) (40). This combination of nutrients was used as a base to which HMOs were added to simulate breastfeeding. HMOs were added as a mixture of five abundant HMOs in human milk (5HMO-Mix), 2′-FL, 3-FL, LNT, 3′-SL, and 6′-SL (Novonesis, Denmark) in the ratios 52:13:26:4:5, reflecting average physiological concentrations (5.75 g L^−1^) as previously described (35). Infant digestibility indices were used to calculate the fractions of macronutrients and HMOs that pass from the small intestine into the colon: 2% lactose (41), 2% casein (42), 20% whey (43), and 99% HMOs (44).

As a result, SIIEM consisted of (g L^−1^ demineralized H_2_O): 25.5 lactose, 1.7 casein, 28.9 whey (MyProtein), 0.1 ox-bile, 0.8 CaCl_2_·2H_2_O, 0.01 FeSO_4_·7H_2_O (GC Chemikalien), 0.004 hemin, 4.7 K_2_HPO_4_, 4.5 KCl, 0.75 MgSO_4_·7H_2_O, 4.0 mucin (porcine type II), 2.5 yeast extract (Thermo Fisher Scientific), 8.4 NaCl, 1.5 NaHCO_3_, 4.5 peptone, 0.8 cysteine HCl, 10.0 polysorbate 80 (Tween 80), and 1.5 mL vitamin solution consisting of (mg L^−1^ H_2_O): 1 menadion, 2 biotin, 0.5 vitamin B12, 10 pantothenic acid, 5 nicotinamide, 5 para-aminobenzoic acid, and 4 thiamine. All chemicals were purchased from Sigma-Aldrich unless stated otherwise. To prepare SIIEM-HMO, HMOs were added to SIIEM at a concentration of 101.9 g L^−1^ corresponding to 6.12 g HMOs per day, based on average feeding volumes of 4- to 6-month-old infants.

Proteins and HMOs were pasteurized (20 min, 72°C), lactose and vitamin solutions were filter sterilized (0.2 µm filter), and the remaining chemicals were autoclaved (15 min, 121°C). The final pH was adjusted to 5.8.

The dialysate solution consisted of (g L^−1^ demineralized H_2_O): 2.5 K_2_HPO_4_·3H_2_O, 4.5 NaCl, 0.005 FeSO4·7H_2_O, 0.01 ox-bile, 0.5 MgSO_4_·7H_2_O, 0.45 CaCl_2_·2H_2_O, and 0.4 CyHCl) and 1 mL of the vitamin solution. The final pH was adjusted to 5.8.

### Experimental setup

The frozen fecal-dialysate mixtures were thawed and homogenized in an anaerobic chamber (90% N_2_, 5% H_2_, 5% CO_2_) at 37°C. Multiple samples from the same infant were pooled. The number of samples used in each experiment, depended on the weights of the fecal samples from the individual infants. The homogenized mixture was used to inoculate the system and individual compartments were filled with dialysate solution up to 120 mL. Prior to inoculation, the compartments were flushed with nitrogen for at least 3 h.

**Fig 1:**
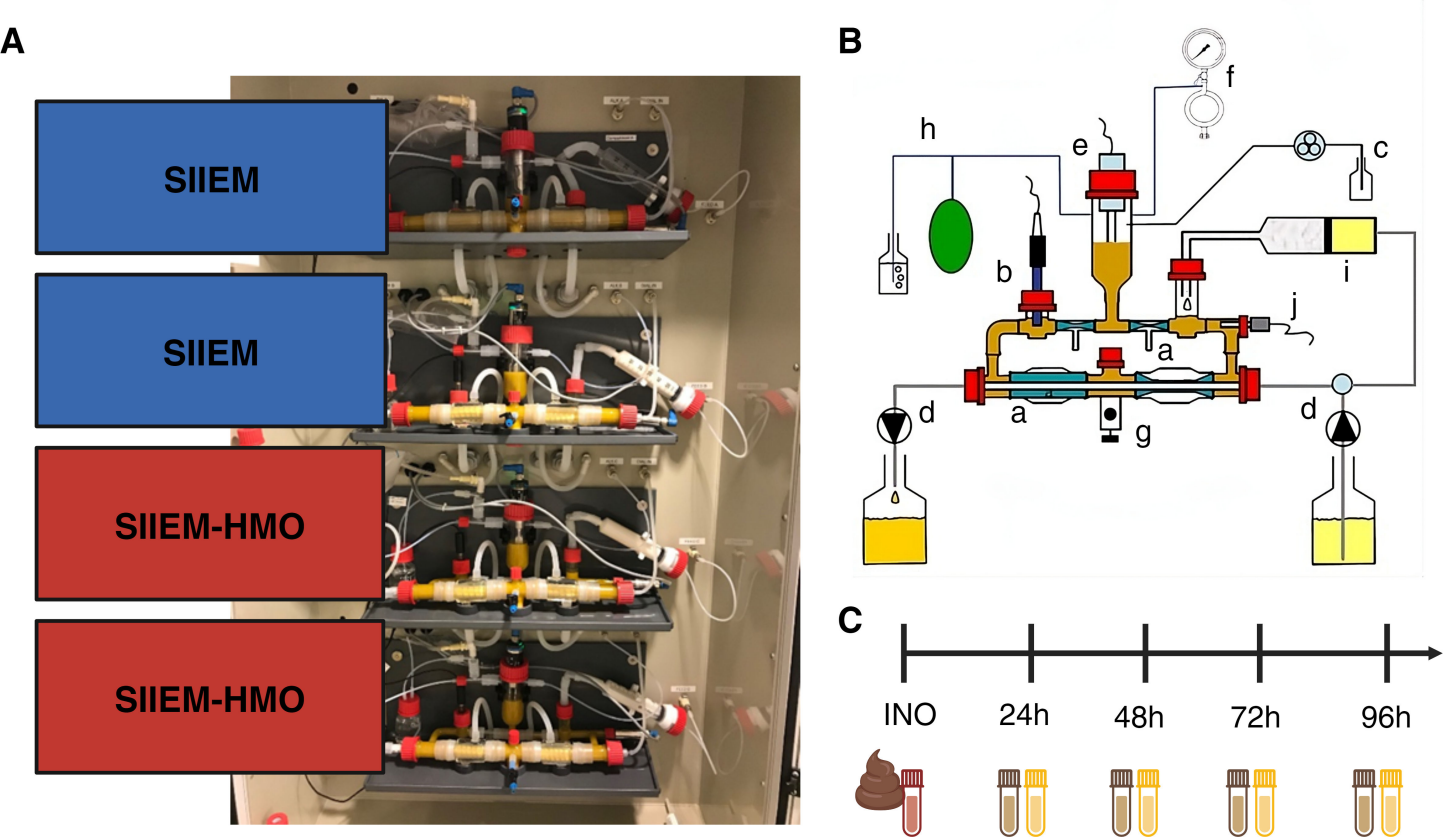
Infant TIM-2. (**A**) Experimental setup. In practice the order of the compartments was randomized in each experiment. (**B**) Schematic representation of one I-TIM-2 compartment (created by and reproduced with permission from the TIM Company) (**a**) peristaltic compartments with a dialysate membrane inside, (**b**) pH electrode, (**c**) NaOH inlet, (**d**) dialysate system, (**e**) level sensor, (**f**) N_2_ inlet, (**g**) sampling port, (**h**) gas outlet, (**i**) feeding syringe, and (**j**) temperature sensor. (**C**) Sampling overview. The donor feces (INO) were sampled before inoculation, while the lumen and the dialysate of I-TIM-2 were sampled at 24, 48, 72, and 96 h.

Each experimental run consisted of four independent compartments operating in parallel (Fig. 1A). Two received SIIEM and two SIIEM-HMO at 0.04 mL min^−1^, while the dialysate rate was set at 1.5 mL min^−1^. The temperature was maintained at 37°C and the pH at 5.8 (Fig. 1B).

Samples were taken before inoculation from the mixture that was used to inoculate the system (INO) and subsequently from the compartments (simulated lumen) and dialysate (collection of molecules, e.g., metabolites removed from the compartments via passive diffusion through the membranes) at 24, 48, 72, and 96 h (Fig. 1C) and stored at −80°C. Twice a day, 12.5 mL luminal content was removed from each compartment to simulate passage through the infant colon (45).

### Quantitative real-time PCR

DNA was extracted from 250 mg inoculum and luminal samples using the Qiagen DNA Isolation kit (DNeasy Powerlyzer Powersoil Kit) following the manufacturer’s protocol with minor changes: bead beating was done using a Retsch MM300 beadbeater for 10 min at 30 cycles s^−1^ and centrifugation was performed for 1 min at 13,000 rpm. DNA concentrations were measured using a Qubit 2.0 Fluorometer with dsDNA HS Assay Kit.

Cell counts of subspecies *B. longum* and *B. infantis* were measured using primers, lon_0274_F (GAGGCGATGGTCTGGAAGTT) and lon_0274_R (CCACATCGCCGAGAAGATTC) (46), and Sia-266F (GACGAGGAGGAATACAGCAG) and Sia-676R (CACGAACAGCGAATCATGGATT) (47), respectively. Each reaction was performed in triplicate with 5 µL PCR-grade water, 1.5 µL forward and reverse primers, 10 µL SYBR Green I Master 2× (LightCycler 480 SYBR Green I Master, Roche), and 2 µL template DNA for a final volume of 20 µL. Standard curves were generated from 10-fold dilutions of target bacterial DNA. Plates were run on the QuantStudio 5 with the following program: 5 min preincubation at 95°C followed by 45 cycles of 15 s at 95°C, 15 s at 50°C, and 15 s at 72°C, and a subsequent melting curve analysis of 5 min at 95°C, 1 min at 68°C, and continuous temperature increase until 98°C. Data were analyzed with the QuantStudio 5 Design and Analysis software (v2.7.0).

### Shotgun metagenomic sequencing

DNA was extracted from inoculum and luminal samples. Library prep was performed with 100 ng of DNA per sample using the Nextera DNA Flex Library Prep Kit (Illumina). Sequencing was performed using a NovaSeq 6000 (Illumina) generating >10 million paired end 100 nt reads per sample. Reads were demultiplexed with bcl2fastq Conversion Software (version 2.20; Illumina), and adapters were trimmed with Skewer (48). Taxonomic classification and relative abundance were generated by Unseen Bio Aps as follows: quality control of raw sequencing reads was performed with FastQC (49) and fastp (50). Human DNA was removed by mapping reads with Kraken 2 (51). The remaining reads were mapped to the MGnify human gut microbiome-specific reference database (52) with Kraken 2 and classified at the species level using Bracken (53).

### Short-chain fatty acid quantification

Short-chain fatty acid (SCFA) concentrations were measured by Clinical Microbiomics using a high polarity column (Zebron ZB-FFAP, GC Cap. Column 30 m × 0.25 mm × 0.25 µm) installed in a GC (7890B, Agilent) coupled with a time-of-flight MS (Pegasus BT, LECO). Samples were acidified using HCl, and deuterium labeled internal standards were added. The system was controlled by ChromaTOF (LECO). Raw data were converted to netCDF format using Chemstation (Agilent), before the data were imported and processed in Matlab R2014b (Mathworks, Inc.) using the PARADISe software (54).

### Human milk oligosaccharide quantification

HMO concentrations were measured by Clinical Microbiomics using a Thermo Scientific Vanquish LC coupled to a Thermo Q Exactive HF MS. An electrospray ionization interface, in positive mode, was used as ionization source. The Ultra-Performance Liquid Chromatographer was mounted with a Waters Aquity UPLC BEH Amide (2.1 × 100 mm^2^, 1.7 µm) with acetonitrile/water (0.3:9.7) plus 0.1% formic acid as the aqueous eluent and acetonitrile/water (9.5:0.5) plus 0.1% formic acid as the organic eluent. Peak areas were extracted using Compound Discoverer 3.2 (Thermo Scientific).

### Statistical analyses

All statistical analyses were performed using R Statistical Software v4.1.0 (55). The data were tested for normality using the Shapiro-Wilk test. Where necessary, the data were log10 transformed and the microbiome data specifically were center log-ratio (clr) transformed to account for compositionality (56). Pairwise comparisons between samples from the donor feces and from I-TIM-2 with the two different diets, SIIEM and SIIEM-HMO, were performed using linear mixed-effects models with false discovery rate correction to account for multiple testing (57). Corrected *P*-values < 0.05 were considered statistically significant. Results are shown as mean ± SD.

Shannon diversity was calculated to define alpha diversity with the phyloseq package (58). Beta diversity was calculated using weighted and unweighted Unifrac distances using the vegan package (59) and visualized using Principal coordinates analysis (PCoA) using phyloseq. Permutational analysis of variance (PERMANOVA) (60) was used to calculate the effect sizes of environmental variables on the microbiome using vegan.

## RESULTS

### Infant donor characteristics and fecal microbiome composition

**Fig 2:**
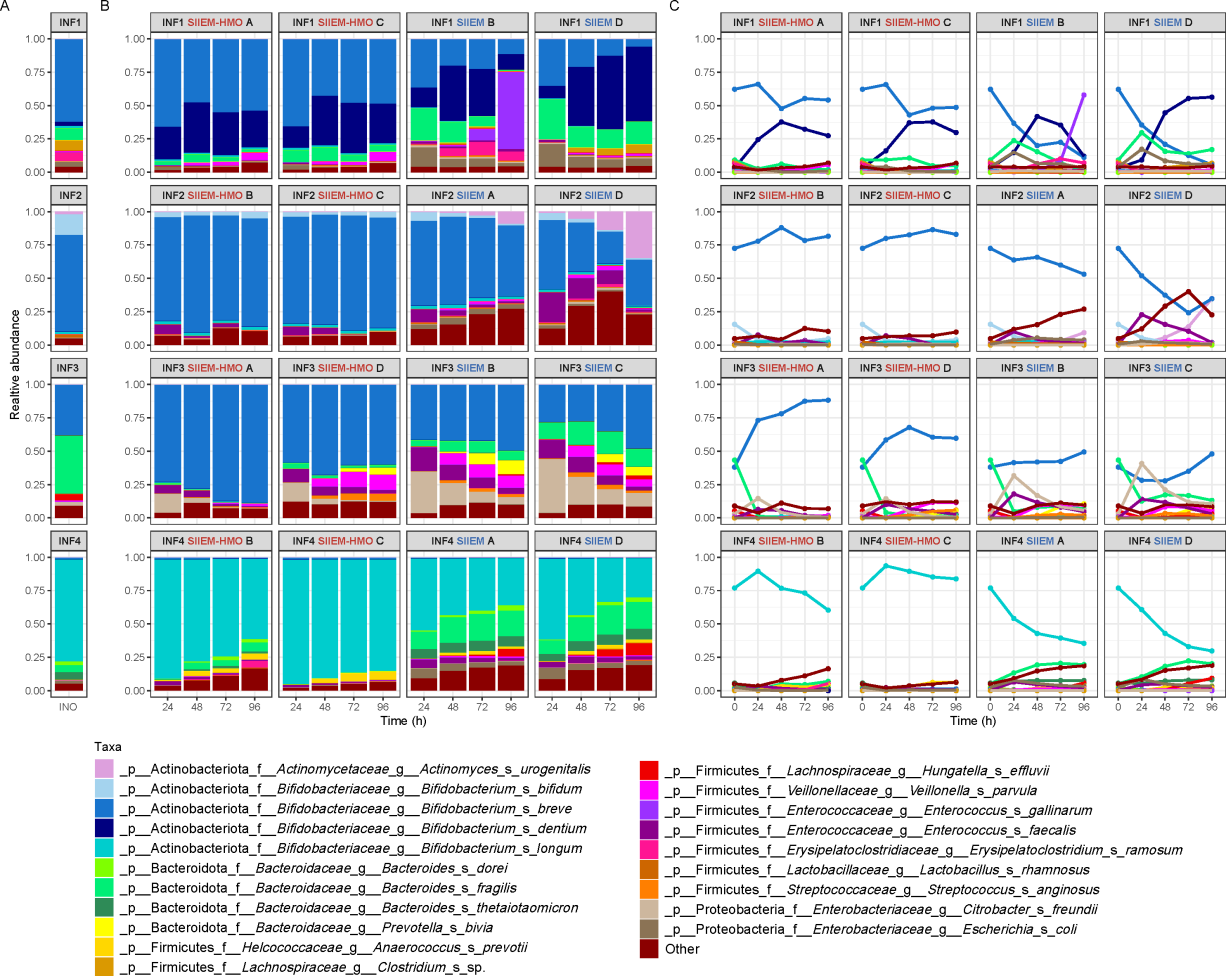
Microbiome composition and dynamics. Relative abundance of the twenty most prevalent bacterial species and the rest grouped as “other”. (**A**) Microbiome composition of the fecal inocula (INO). (**B**) Temporal development of the microbiome composition in I-TIM-2 in the independent experiments with individual donors (INF1-4). (**C**) Complementary line plots to interpret the temporal compositional dynamics.

Donor characteristics are shown in Table 1. The infants included two males and two females that were 2–4 months old. Two were vaginally born and two were delivered by Cesarean section. All four infants were primarily breastfed, two received supplemental formula (mixed fed) and none had started weaning. Their fecal microbiome compositions reflected the primary feeding with human milk, as the donor fecal inocula were dominated by species known to utilize HMOs from the genera *Bifidobacterium* and *Bacteroides* (68.7 ± 22.2% and 19.1 ± 17.1%, respectively) (Fig. 2A), in concordance with previously reported percentages for breastfed and mixed fed infants of similar ages (61). The composition of the HMO degrading communities differed in each infant. INF1 and INF2 were dominated by *B. breve* (62.2% and 72.5%, respectively). INF3 was dominated by *B. fragilis* (44.1%) and *B. breve* (38.0%). INF4 was dominated by species *B. longum* (76.9%), of which 51.2% (qPCR based ratio) was *B. infantis* (Fig. S2A) and 0.10% was subspecies *B. longum* (Fig. S2B).

### I-TIM-2 microbiome composition

#### Inoculum species retained in I-TIM-2

For each donor, a high number of species derived from the inocula (INO) were retained in I-TIM-2 with both diet types (SIIEM 87.5 ± 8.9%; SIIEM-HMO 69.0 ± 12.9%) (Fig. S1). Furthermore, these species constituted a high proportion of the bacterial communities (cumulative relative abundance; SIIEM 85.1 ± 11.7%; SIIEM-HMO 92.4 ± 6.4%) (Fig. S1).

Taxa that were not retained in I-TIM-2 included species of *Haemophilus*, *Clostridium* and *Streptococcus* that may have been environmental, transient colonizers in the donors and/or were not capable of growth in the set conditions of I-TIM-2. Alternatively, species of these genera were retained in I-TIM-2, but decreased below the detection threshold, which is sequencing depth-dependent and can influence data analysis outcome (62).

#### Alpha diversity

**Fig 3:**
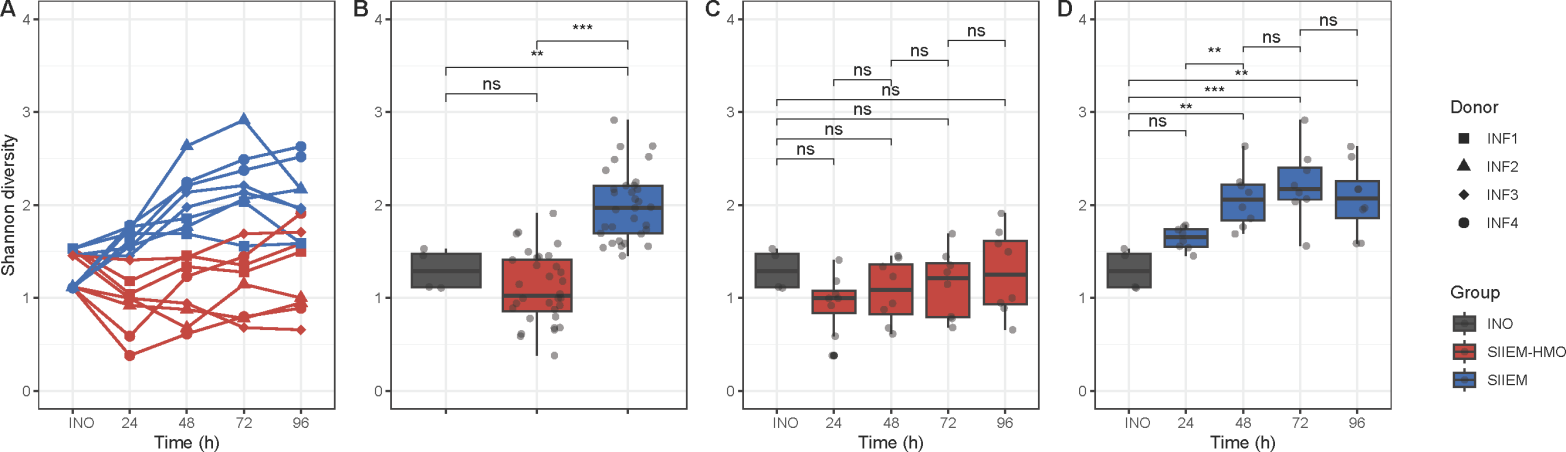
Shannon diversity of the microbial communities. (**A**) Temporal development in I-TIM-2 with SIIEM and SIIEM-HMO in the independent experiments with individual donors (INF1–4). (**B**) Comparisons between samples of the donor fecal inocula (INO) and from I-TIM-2 with SIIEM and SIIEM-HMO. (**C**) Comparisons between samples of INO and from I-TIM-2 with SIIEM-HMO. (**D**) Comparisons between samples of INO and from I-TIM-2 with SIIEM. Results shown from linear mixed-effects models (***P < 0.001, **P < 0.01, *P < 0.05).

The model effectively maintained the species richness and diversity of the donor feces, although the temporal dynamics were diet-dependent (Fig. 3A). Shannon diversity (a combination of species richness and distribution) increased with SIIEM (INO 1.3 ± 0.2 vs SIIEM 2.0 ± 0.4, *P* < 0.01) (Fig. 3D), while it was maintained with SIIEM-HMO (INO 1.3 ± 0.2 vs SIIEM-HMO 1.1 ± 0.4, *P* > 0.05) (Fig. 3C).

#### Beta diversity

**Fig 4:**
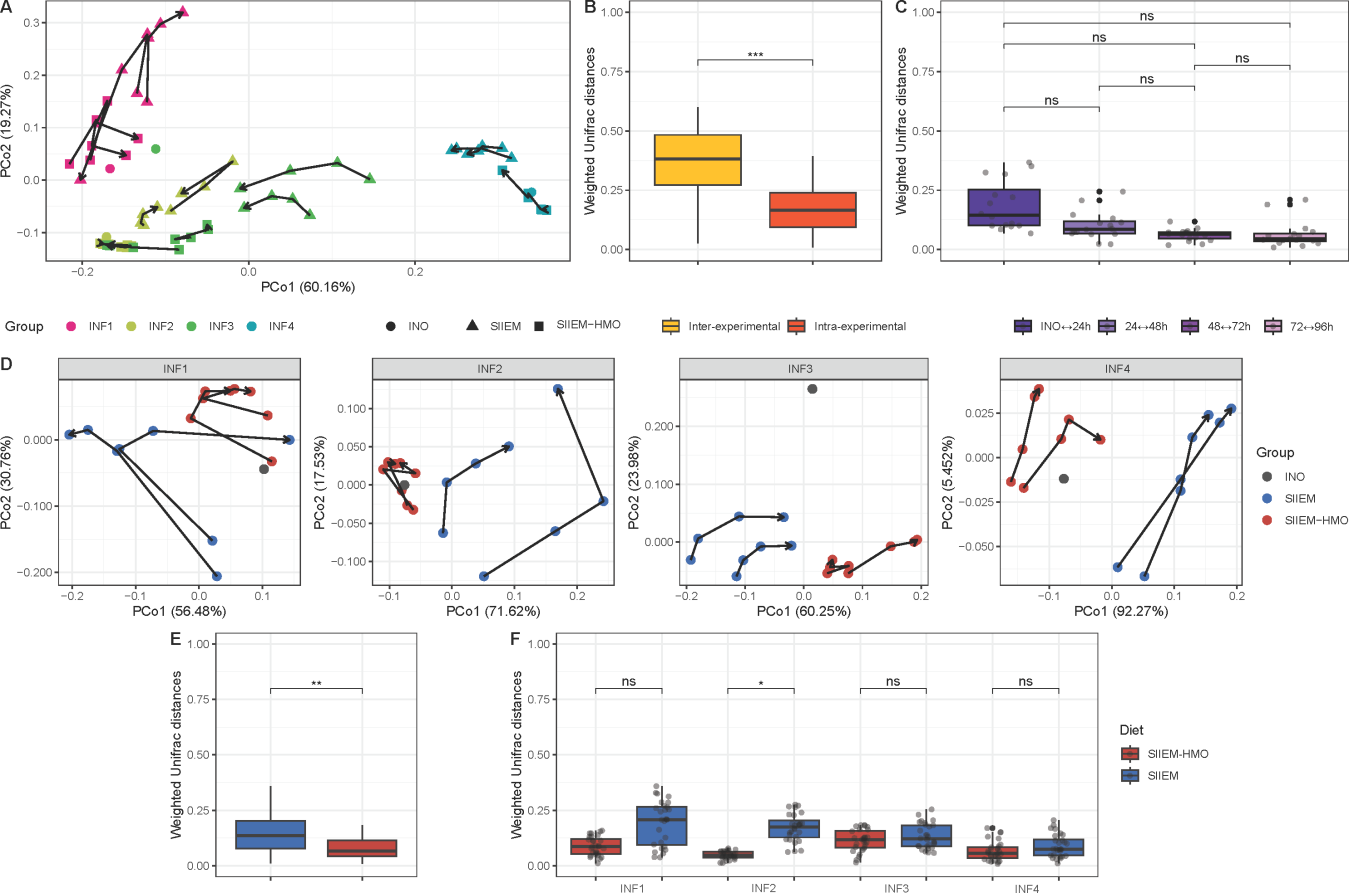
Beta diversity. (**A**) Weighted Unifrac distance (WU) based principal coordinates analysis (PCoA) visualizing compositional differences between samples. Each point represents a microbial community, distances represent differences between microbial communities and arrows connect samples from individual donors (INF1–4) and compartments temporally in sequential order. (**B**) Comparisons of WU between intra- and interexperimental samples. (**C**) Comparisons of WU between samples from different time points. (**D**) WU-based PCoA visualizing differences between samples of the donor fecal inocula (INO), SIIEM, and SIIEM-HMO. (**E**) Comparisons of WU between I-TIM-2 samples with SIIEM and SIIEM-HMO. (**F**) Comparisons within donors of WU between I-TIM-2 samples with SIIEM and SIIEM-HMO. Results shown from linear mixed-effects models (***P < 0.001, **P < 0.01, *P < 0.05).

For each donor, the original INO microbiome composition was preserved in I-TIM-2 throughout the experiments. The greatest compositional variation between samples was caused by the donors (*R*^2^ = 0.70, *P* < 0.001), while time did not significantly cause variation (Fig. 4A). This was determined using PERMANOVA, which associated the microbiome composition with the environmental factors based on (dis)similarities quantified by the distance metric, Weighted Unifrac (WU) (a measure based on the amounts of shared species, their distribution and relatedness).

The system was stable as the microbiome composition of related, intra-experimental samples was more similar compared to unrelated, inter-experimental samples (0.17 ± 0.09 vs 0.37 ± 0.12 WU, respectively, *P* < 0.001) (Fig. 4B). Also, the compositional differences between intra-experimental samples were not significant over time (0.13 ± 0.09 WU) (Fig. 4C).

The effect of the HMOs on the microbiome composition was strongly donor-dependent (*R*^2^ = 0.15, *P* < 0.05, *R*^2^ = 0.30, *P* < 0.01, *R*^2^ = 0.21, *P* < 0.01, *R*^2^ = 0.54, *P* < 0.01, INF1-4, respectively) (Fig. 4D). The composition was more stable with HMOs, as the microbial communities were more similar with SIIEM-HMO compared to SIIEM (0.17 ± 0.09 vs 0.10 ± 0.07 WU, *P* < 0.01, respectively) (Fig. 4E and F), and were compositionally more similar to their respective INO (0.24 ± 0.07 vs 0.17 ± 0.1 WU, respectively, *P =* 0.10) (Fig. 4D).

The system was highly reproducible as replicate compartments developed similarly over time (Fig. 2B and C). Nevertheless, one SIIEM compartment from INF1 at *t* > 72 h had growth of *Enterococcus gallinarum*, which is a facultative anaerobe (62). Thus, we speculate that in this one occasion oxygen might have entered the compartment (Fig. 2B and C).

#### Relative and absolute abundance of key bacterial taxa

With SIIEM, the relative abundance of HMO utilizing bifidobacteria, *B. bifidum*, *B. breve*, and *B. longum*, decreased compared to INO, with concomitant donor-dependent increases in other species such as *Bacteroides fragilis*, *Escherichia coli*, *Veillonella parvula*, *Enterococcus faecalis*, *Prevotella bivia*, and *Citrobacter freundii* (Fig. 2B and C). Instead, SIIEM-HMO preserved the relative abundance of bifidobacteria compared to INO (Fig. 2).

We were unable to distinguish between subspecies *B. longum* and *B. infantis* using shotgun metagenomics (63). These subspecies differ considerably in their metabolic capabilities. For instance, strains of *B. longum* utilize many types of plant-derived carbohydrates (64) and only few types of HMOs (LNT and LNnT) (65), while strains of *B. infantis* utilize many types of HMOs (66). Therefore, we used a subspecies-specific qPCR to distinguish between them. Samples from INF2 contained *B. longum* (log_10_; SIIEM 6.8 ± 0.2; SIIEM-HMO 6.4 ± 0.2), while samples from INF4 contained *B. longum* (log_10_; SIIEM 6.7 ± 0.2; SIIEM-HMO 6.1 ± 0.2) and *B. infantis* (log_10_; SIIEM 9.4 ± 0.4; SIIEM-HMO 9.5 ± 0.3) (Fig. S2).

### HMO utilization

**Fig 5:**
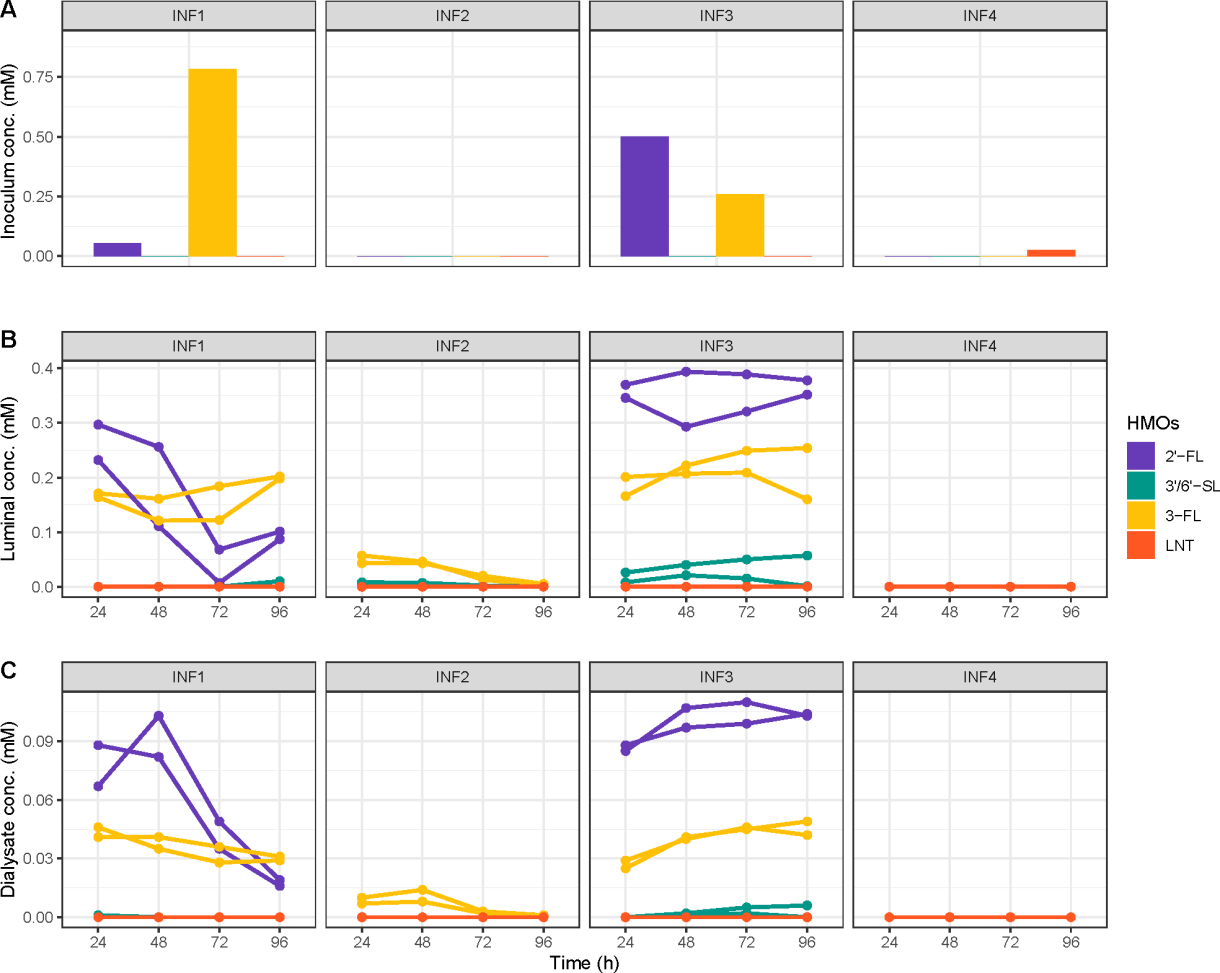
Human milk oligosaccharide (HMO) concentrations (**A**) in the donor fecal inocula (INO). (**B**) Temporal dynamics in I-TIM-2 compartments (simulated lumen) with SIIEM-HMO in the independent experiments with individual donors (INF1–4). (**C**). Temporal dynamics in the dialysate (collection of molecules that passively diffuse through the membranes).

The added HMOs were continuously distributed throughout the compartments via peristaltic movements and measured at each time point within the compartments (simulating the lumen) and in the dialysate (collection of molecules that passively diffuse through the membranes). The composition of HMOs was similar in the lumen and dialysate. Importantly, the profiles of HMO utilization of the respective donors were replicated in I-TIM-2 (Fig. 5) and reflected the different HMO utilizing bacterial communities. LNT was completely used up in all samples, while utilization of the other HMOs was donor-dependent. For instance, 2′-FL and 3-FL accumulated in samples from INF1, while 3′-SL and 6′-SL were entirely utilized. These samples contained mostly *B. breve* (relative 53.6 ± 8.5% relative abundance) (Fig. 2) that utilizes LNT (67), *B. dentium* (30.3 ± 7.6%) of which some strains utilize LNT (68), and *B. fragilis* (5.4 ± 3.2%) that uses all the supplied HMOs, but prefers and more efficiently utilizes sialylated HMOs (28, 69).

Nearly all HMOs were utilized in samples from INF2. They contained *B. bifidum* (3.3 ± 0.9%) and *B. breve* (82.2 ± 3.6%). The former degrades all the supplied HMOs extracellularly and releases the degradation products into the surrounding environment (70) for potential cross-feeding with *B. breve* (71). In INF3 only LNT was entirely utilized. These samples were dominated by *B. breve* (71.6 ± 12.1%), which generally only utilizes this HMO. In INF4 all HMOs were utilized. These samples were dominated by species *B. longum* (81.5 ± 10.9%), of which the majority was *B. infantis* (based on the subspecies-specific qPCR) (Fig. S2) that can efficiently utilize all the supplied HMOs (66).

### Metabolic output

**Fig 6:**
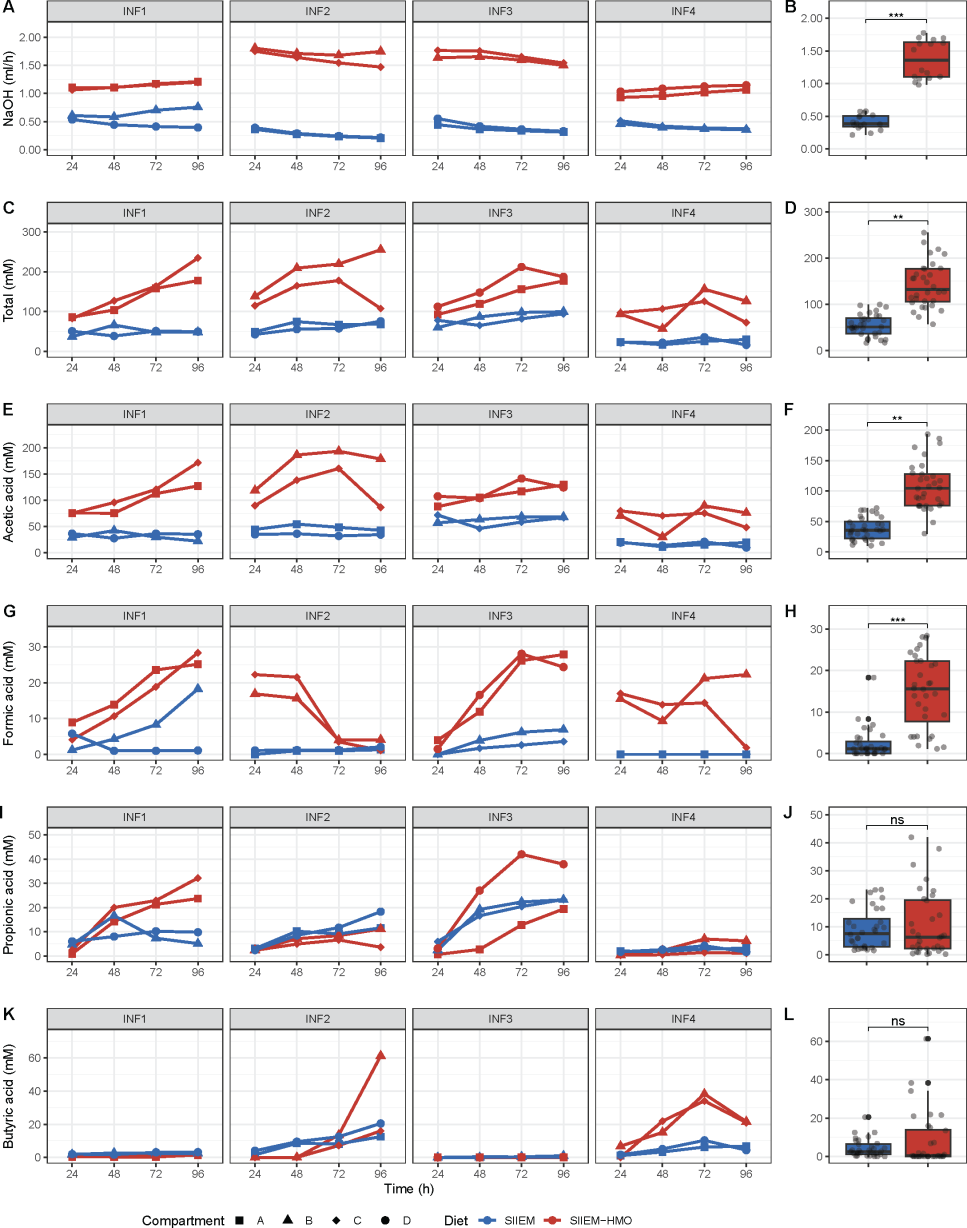
Temporal dynamics of I-TIM-2 NaOH consumption and short-chain fatty acid (SCFA) production within the compartments (simulated lumen). (**A**) NaOH consumption with SIIEM and SIIEM-HMO in the separate experiments with individual donors (INF1-4). (**B**) Comparison of NaOH consumption between I-TIM-2 samples with SIIEM and SIIEM-HMO. Production of (**C**) Total SCFAs (acetic, formic, propionic and butyric acid), (**E**) Acetic acid, (**G**) Formic acid, (**I**) Propionic acid and (**K**) Butyric acid. Comparisons of (**D**) Total SCFAs, (**F**) Acetic acid, (**H**) Formic acid, (**J**) Propionic acid and (**L**) Butyric acid between I-TIM-2 samples with SIIEM and SIIEM-HMO. Results shown from linear mixed-effects models (***P < 0.001, **P < 0.01, *P < 0.05).

**Fig 7:**
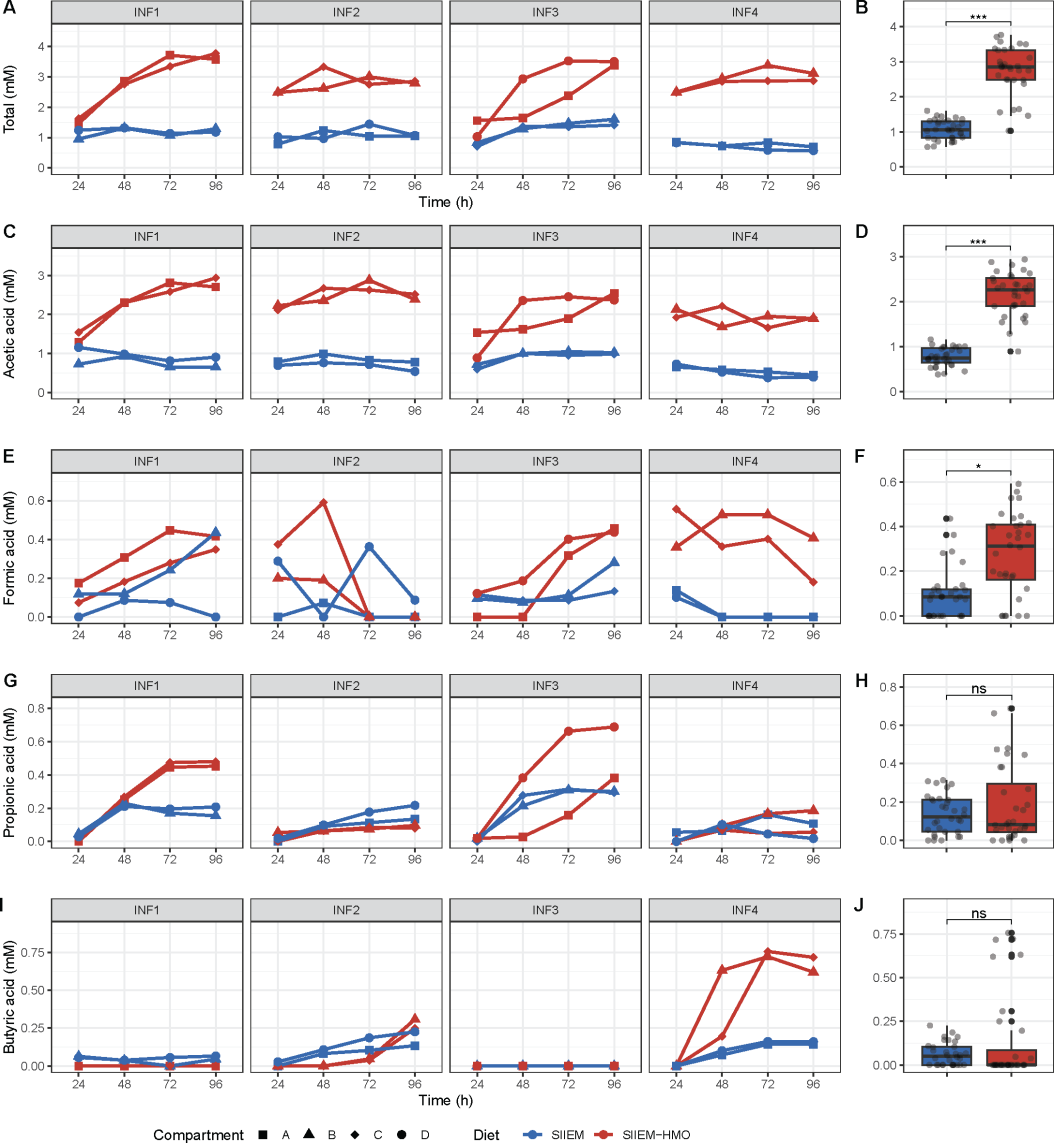
Temporal dynamics of I-TIM-2 short-chain fatty acid (SCFA) concentration in the dialysate (collection of molecules that passively diffuse trough the membranes). Concentration of (**A**) Total SCFAs (acetic, formic, propionic and butyric acid), (**C**) Acetic acid, (**E**) Formic acid, (**G**) Propionic acid and (**I**) Butyric acid. Comparisons of (**B**) Total SCFAs, (**D**) Acetic acid, (**F**) Formic acid, (**H**) Propionic acid and (**J**) Butyric acid between I-TIM-2 samples with SIIEM and SIIEM-HMO. Results shown from linear mixed-effects models (***P < 0.001, **P < 0.01, *P < 0.05).

In I-TIM-2 NaOH consumption was used as a proxy for overall acid production by the microbial communities. The consumption rate was higher with SIIEM-HMO compared to SIIEM (1.4 ± 0.3 ml h^−1^ vs 0.4 ± 0.1 ml h^−1^, *P* < 0.001) (Fig. 6A and B). In concordance, total SCFA (acetic acid, formic acid, propionic acid and butyric acid) production was higher with SIIEM-HMO compared to SIIEM in both the lumen and dialysate (lumen 142.0 ± 49.7 vs 54.1 ± 24.0 mM, *P* < 0.01, dialysate 29.7 ± 7.4 vs 11.5 ± 2.9 mM, *P <* 0.001, respectively) (Fig. 6C and D) (Fig. 7A and B). Overall, the temporal dynamics of SCFA production were similar in the lumen and dialysate.

Within donors total luminal SCFA concentrations in I-TIM-2 were donor and diet-dependent and ranged from 16.2 to 255.2 mM in concordance with fecal SCFA concentrations in breastfed and mixed fed infants of the same age (72).

The concentrations of acetic and formic acid were higher in samples with SIIEM-HMO compared to SIIEM (acetic acid, lumen 108.0 ± 39.7 vs 38.2 ± 18.2 mM, *P <* 0.01, dialysate 23.3 ± 5.4 vs 8.3 ± 2.1 mM, *P <* 0.001; formic acid lumen 15.0 ± 8.7 vs 2.3 ± 3.7 mM, *P <* 0.001, dialysate 3.0 ± 2.0 vs 1.1 ± 1.2 mM, *P <* 0.05) (Fig. 6E through H) (Fig. 7C through F).

Additionally, in INF1 and INF3 propionic acid was produced (Fig. 6I, J, 7G and H). These communities contained propionate producing species *B. fragilis* (73) and *V. parvula* (74) (INF1 10.6 ± 8.4% and 1.9 ± 2.1%; INF3 6.3 ± 6.1% and 5.6 ± 4.8%, respectively) (Fig. 2B and C). In INF2 and INF4 both propionic and butyric acid were produced (Fig. 6I through L) (Fig. 7G through J). INF2 contained propionate producers *Bacteroides caccae* (75) and *V. parvula* (4.2 ± 4.4% and 2.0 ± 1.6%, respectively) and butyrate producers *Anaerococcus hydrogenalis* and *Anaerococcus obesiensis* (0.3 ± 0.6% and 0.3±0.6%, respectively) (76), whereas INF4 contained propionate producers *B. fragilis*, *B. thetaiotaomicron*, and *V. parvula* (10.4 ± 9.1%, 4.0 ± 3.5%, and 0.6 ± 0.5%, respectively) and butyrate producer *Anaerococcus prevotii* (2.5 ± 1.9%) (76).

## DISCUSSION

We developed and validated an *in vitro* model simulating the infant colonic environment, I-TIM-2, based on the TIM-2, a system with unique features such as simulated peristalsis and removal of metabolites via passive diffusion through membranes (31).

Several *in vitro* colonic models have been developed or adapted to simulate infant conditions (14–20). These are challenged by the limited availability of infant fecal material (45) that is additionally relatively low in microbial density, diversity, and functional redundancy (77). Biodiversity and functional redundancy contribute positively to the stability and persistence of ecological communities (78, 79). Thus, the infant gut microbiome is more sensitive to perturbations and changes in composition when encountering disturbances such as the introduction to an artificial *in vitro* colonic environment.

To increase compositional stability, researchers have pooled samples from different infant donors (14, 20), selected suitable donors based on preceding short-term fermentation experiments (80–82) and used continuous inoculation from reactors containing an immobilized microbiota (82–85).

However, pooling samples from different infants eliminates the interindividual differences in microbiome composition. It retains the overall functional properties, but causes differences in the metabolic output compared to the individual infant (86, 87), and undermines the high interindividual variation observed particularly in the infant population.

The I-TIM-2 experiments required high amounts (>60 g) of fecal material, which exceeded the average amount of a single infant stool sample (45). Therefore, we pooled samples from the same infant (collected over 6 weeks, while the infants received the same diet), which introduced the least amount of variation possible. This allowed us to retain the distinct features of the microbiome of each infant, and it minimized the risk of losing taxa due to sample handling and storage (88)

Fecal samples from four infants representing different microbial compositions were selected to study the effects of the I-TIM-2 conditions and HMO supplementation on various microbial communities and their metabolic output. However, it should be acknowledged that the limited number of samples studied derive from a restricted subset of the Danish infant population. Extrapolation to the general infant population should therefore be done with caution. Nonetheless, the microbial composition of the four infants selected, dominated by specific species within *Bifidobacterium* and *Bacteroides*, resemble the range of dominating taxa found in the general breastfed infant population (29, 89).

The main characteristics of each of the infant donor fecal bacterial communities were retained in I-TIM-2 including high bacterial density, alpha diversity, and key bacterial taxa. The system was fed with two types of diets (SIIEM and SIIEM-HMO), which influenced the developing communities. SIIEM, simulating formula feeding, increased alpha diversity with increases in relative and absolute abundance of species like *B. fragilis*, *E. coli*, *V. parvula*, *E. faecalis*, *P. bivia*, and *C. freundii*. This is in concordance with reported effects on the infant fecal microbiome, where exclusive formula feeding was also associated with higher alpha diversity and increased prevalence and abundance of the abovementioned genera (90–92).

The specific species that increased in abundance were donor-dependent. Some of these species were below the detection threshold in the respective inocula and appeared only after >24 h in the model, indicating that they were selected for under the I-TIM-2 conditions without HMOs. These species replaced bifidobacteria with known health effects on the host and instead introduced compositional changes with unknown effects. For example, species of *Enterococcus* and *Escherichia* comprise a wide variety of strains that range from commensals to severe pathogens depending on the presence of virulence factors that are frequently encoded on genetic elements (93–95), thus transferable between strains.

The addition of physiologically relevant concentrations of HMOs, promoted the HMO utilizing species, *B. bifidum*, *B. breve*, *B. longum*, *B. infantis*, *B. fragilis*, *B. thetaiotaomicron*, and *B. dorei.* The HMOs preserved the high relative abundance of these species in proportions similar to the donor feces, without the increase of other genera. Thus, the community dynamics were maintained within I-TIM-2 with the addition of HMOs and the resulting bifidobacterial dominance likely prevented the colonization of other microbes (96).

The effects of the 5HMO-Mix were recently evaluated in a randomized, controlled clinical study (36). Here, the fecal microbiome of infants that received infant formula supplemented with this HMO mix was compared to infants fed the same infant formula without HMOs and to a breastfed reference group. The 5HMO-Mix promoted microbial communities that resembled those of the breastfed infants with higher relative abundance of bifidobacteria and decrease in other genera compared to the infants fed control formula. Our study mirrored these effects, which demonstrates that the compositional dynamics of I-TIM-2 are comparable to microbiome compositions observed *in vivo*.

I-TIM-2 retained the HMO degradation capabilities of the fecal bacterial communities, as HMO utilization profiles of the donor feces and *in vitro* samples were highly similar. The added 5HMO-Mix consisted of five of the most simple and abundant HMOs in human milk, 2′-FL, 3-FL, LNT, 3′-SL, and 6′-SL, in physiological concentrations and proportions (35). This mixture was sufficient to preserve the composition and metabolic output of the HMO-degrading communities of the donor feces.

I-TIM-2 accurately reproduced the metabolic activity of the infant microbiome as the production of SCFAs aligned with previously reported *in vivo* data. Luminal SCFA concentrations ranged from ~18 to ~226 mM. Such large variations have previously been reported in fecal samples from 1- to 6-month-old, healthy infants, where SCFA concentrations ranged from 29.3 to 894.8 mM (72). In concordance with the different microbial communities connected with their distinct HMO utilization profiles, each community produced different SCFA profiles. All produced acetic acid that increased with the addition of HMOs, while formic acid was produced in all communities with HMOs. Both metabolites are characteristic end products of bifidobacterial fermentation (97). Interestingly, the concentration of propionic and butyric acid produced partly through cross-feeding on acetate (98) differed between donors, due to the differences in the prevalence of propionate and butyrate-producing species. For instance, samples from INF1 and INF3 contained propionic acid in concordance with the presence of known propionate-producing species *B. fragilis* and *V. parvula* (73, 74). Samples from INF2 and INF4 contained propionate and butyrate in concordance with the prevalence of propionate producers *B. fragilis* and *V. parvula* and butyrate producers *A. hydrogenalis*, *A. obesiensis,* and *A. prevotii* (99).

In conclusion, we developed and validated an infant *in vitro* colonic model, I-TIM-2, that accurately reproduced and sustained the microbiome composition and metabolic output of fecal samples from four healthy, breastfed infant donors. This was achieved throughout the experimental runs that lasted 96 h, which makes the I-TIM-2 an ideal system to assess the effects of various modulating strategies on the infant microbiome composition and activity over time.

## Data Availability

Shotgun metagenomics sequencing data were deposited in the Sequence Read Archive (SRA) under BioProject PRJNA1159139.

## SUPPLEMENTAL MATERIAL

Figures S1 and S2 spectrum.00724-24-s0001.pdf Fig. S1: Inoculum derived species in I-TIM-2. Fig. S2: qPCR based log_10_ quantification of subspecies.

ASM does not own the copyrights to Supplemental Material that may be linked to, or accessed through, an article. The authors have granted ASM a non-exclusive, world-wide license to publish the Supplemental Material files. Please contact the corresponding author directly for reuse.
