# Supplementary material for: Development of an infant colon simulating in vitro model, I-TIM-2, to study the effects of modulation strategies on the infant gut microbiome composition and function
Source: Microbiol Spectr. 2024 Oct 8;12(11):e00724-24. doi: 10.1128/spectrum.00724-24 (PMC11537066; doi:10.1128/spectrum.00724-24)
Supplement: Figures S1 and S2 — Fig. S1: Inoculum derived species in I-TIM-2. Fig. S2: qPCR based log10 quantification of subspecies. [file spectrum.00724-24-s0001.pdf]

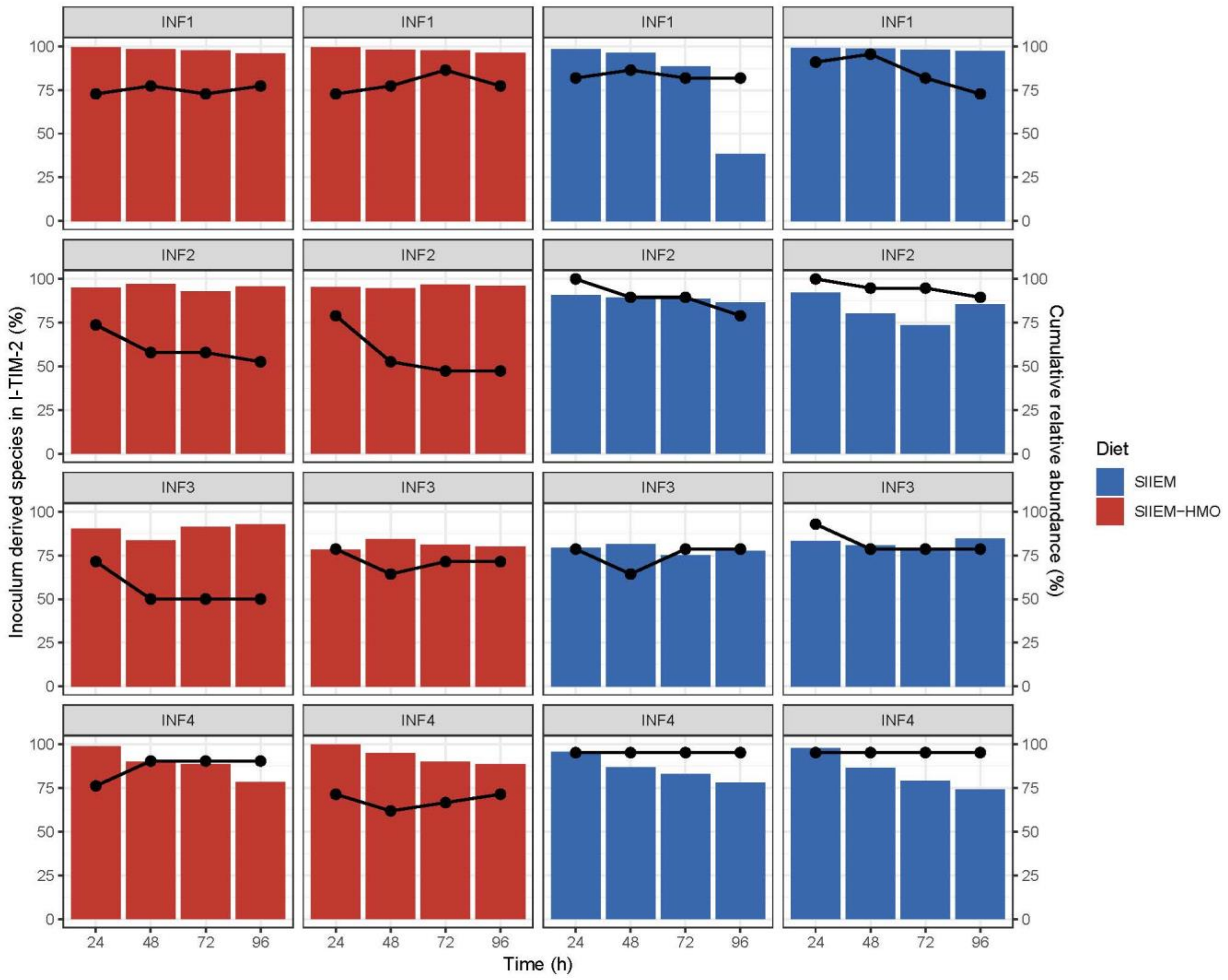

Supplementary figure 1 Inoculum derived species in I-TIM-2. Proportion of species present in the inoculum (abundance >0.01%) that were recreated in I-TIM-2 (>0.01%) shown in lines and their cumulative relative abundance shown in bars with SIIEM and SIIEM-HMO in the independent experiments with individual donors (INF1-4).

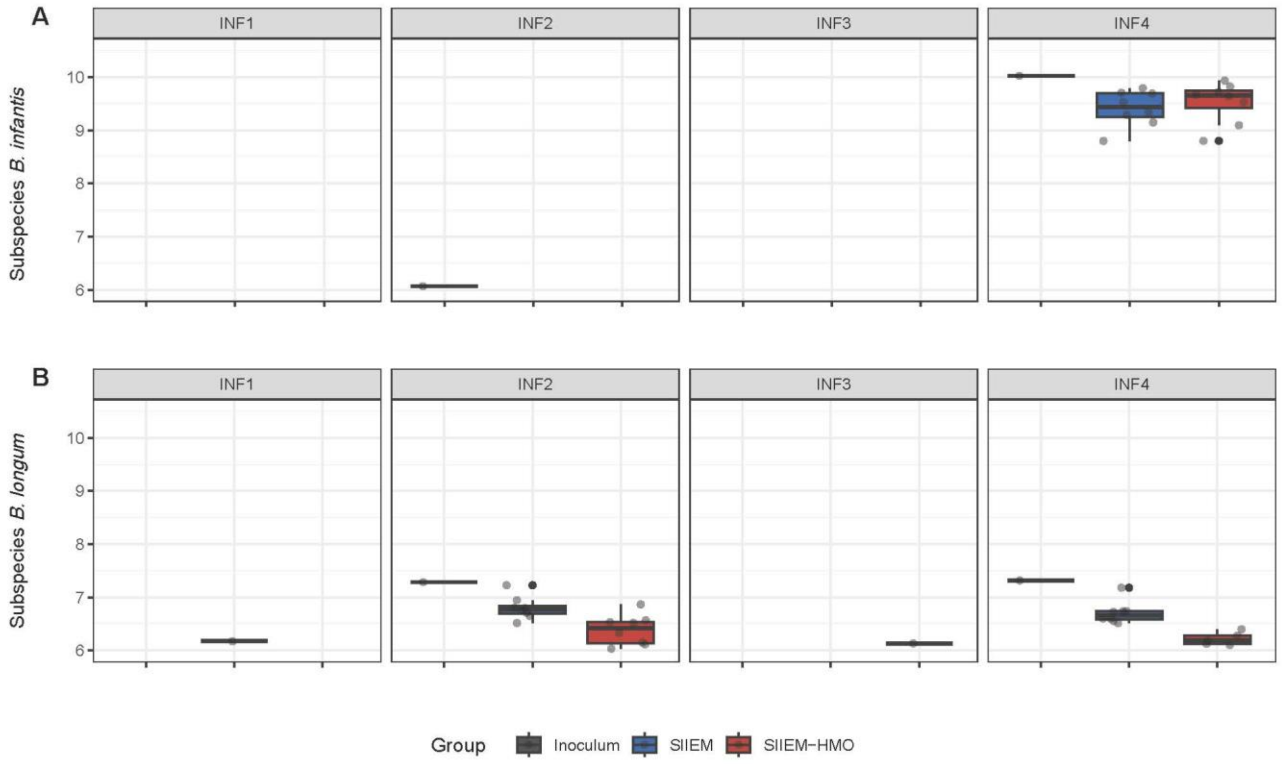

Supplementary figure 2 qPCR based $\log_{10}$ quantification of subspecies (A) B. infantis and (B) B. longum with SIIEM and SIIEM-HMO in the independent experiments with individual donors (INF1-4).
